# Toward a systems-level view of dynamic phosphorylation networks

**DOI:** 10.3389/fgene.2014.00263

**Published:** 2014-08-15

**Authors:** Robert H. Newman, Jin Zhang, Heng Zhu

**Affiliations:** ^1^Department of Biology, North Carolina Agricultural and Technical State UniversityGreensboro, NC, USA; ^2^Department of Pharmacology and Molecular Sciences, Johns Hopkins University School of MedicineBaltimore, MD, USA; ^3^The Solomon H. Snyder Department of Neuroscience, Johns Hopkins University School of MedicineBaltimore, MD, USA; ^4^Department of Oncology, Johns Hopkins University School of MedicineBaltimore, MD, USA; ^5^Department of Chemical and Biomolecular Engineering, Johns Hopkins University School of MedicineBaltimore, MD, USA; ^6^High-Throughput Biology Center, Institute for Basic Biomedical Sciences, Johns Hopkins UniversityBaltimore, MD, USA

**Keywords:** protein phosphorylation networks, protein microarrays, fluorescent biosensors, phosphoproteomics, systems biology, kinase-substrate relationship, cell signaling and regulation, quantitative mass spectrometry

## Abstract

To better understand how cells sense and respond to their environment, it is important to understand the organization and regulation of the phosphorylation networks that underlie most cellular signal transduction pathways. These networks, which are composed of protein kinases, protein phosphatases and their respective cellular targets, are highly dynamic. Importantly, to achieve signaling specificity, phosphorylation networks must be regulated at several levels, including at the level of protein expression, substrate recognition, and spatiotemporal modulation of enzymatic activity. Here, we briefly summarize some of the traditional methods used to study the phosphorylation status of cellular proteins before focusing our attention on several recent technological advances, such as protein microarrays, quantitative mass spectrometry, and genetically-targetable fluorescent biosensors, that are offering new insights into the organization and regulation of cellular phosphorylation networks. Together, these approaches promise to lead to a systems-level view of dynamic phosphorylation networks.

## Introduction

Protein phosphorylation, mediated by protein kinases and opposed by protein phosphatases, is one of the most widespread regulatory mechanisms in eukaryotes. Inside the cell, protein kinases, phosphatases, and their respective substrates are organized into complex phosphorylation networks that govern nearly all cellular processes. In order to achieve signaling specificity in response to a given environmental stimulus, these networks must be precisely coordinated in cellular space and time. This involves regulation at several different levels, including at the levels of protein expression (e.g., enzyme levels), substrate selection, and spatiotemporal regulation of enzymatic activity. Not surprisingly, disruption of these regulatory mechanisms has been implicated in many pervasive diseases, including cancer (Guha et al., 2008; Deschenes-Simard et al., 2014), diabetes (Guo, 2014; Mackenzie and Elliott, 2014; Ortsater et al., 2014), and heart disease (Kooij et al., 2014; Sciarretta et al., 2014). As a consequence, much effort has been dedicated to understanding how these parameters are controlled inside the cell.

In this review, we will examine several methodologies used to study the organization and regulation of cellular phosphorylation networks. Our discussion will be organized around two central questions: (1) which cellular proteins are phosphorylated by a given kinase (or dephosphorylated by a given phosphatase) under physiological conditions and (2) how are specific kinases and phosphatases—and ultimately the signaling networks of which they are a part—regulated within the native cellular environment? We will begin by first discussing the properties of protein phosphorylation that make this post-translational modification an attractive chemical signal for cellular information transfer. We will then highlight some of the traditional methods that researchers have used to study the phosphorylation status of cellular proteins before turning our attention to several emerging technologies that are beginning to provide a dynamic and systems-level view of phosphorylation networks. Together, these approaches promise to shed new light on the organization and regulation of cellular phosphorylation networks.

### Cellular and molecular consequences of phosphorylation

Protein phosphorylation describes the covalent attachment of a negatively charged phosphate group to one of several amino acid residues. Though several reports suggest that phosphorylation of His residues (resulting in the formation of an acid labile phosphoramidate species) may account for up to 10% of protein phosphorylation in some eukaryotes (Klumpp and Krieglstein, 2002; Besant and Attwood, 2010), it is generally believed that the predominant sites of phosphorylation in mammalian proteins are on Ser (~86%), Thr (~12%), and Tyr (~2%) residues (Hunter, 1996). Phosphorylation of these hydroxyamino acids results in the formation of phosphate monoesters that are surpassingly stable under physiological conditions, exhibiting an estimated half-life of 1.1 × 10^12^ years in aqueous solution (Lad et al., 2003). Despite their extremely high stability, phosphate monoesters are nonetheless rapidly hydrolyzed through the catalytic action of protein phosphatases, thus making protein phosphorylation a highly dynamic and “regulatable” post-translational modification inside cells. Importantly, the chemical properties of phosphate monoesters confer unique characteristics to phosphorylated amino acids that are critically important for their role in cellular signal transduction (Dissmeyer and Schnittger, 2011). For instance, a phosphate monoester contains two ionizable oxygens which exhibit pK_a_'s of ~2.2 and 5.8, respectively, (Hunter, 2012). Therefore, depending on its chemical context, the transferred phosphate group is expected to be either dianionic or partially dianionic at physiological pH. Such a high charge density makes phosphate groups well-suited to mediate intra- or intermolecular interactions, such as hydrogen bonds and salt bridges, which can impact protein function.

In most cases, phosphorylation is believed to induce conformational changes in the target protein that influence its behavior inside the cell. For example, phosphorylation-dependent conformational changes can alter the activity of enzymes either directly, by promoting the reorganization of the enzyme active site, or indirectly, through allosteric effects on regulatory domains or subunits (Hunter, 2012). These modes of regulation are particularly important in the coordination of kinase signaling cascades that underlie cellular phosphorylation networks. For instance, recent evidence suggests that phosphorylation of residues within the activation loop, a highly conserved region present in all eukaryotic protein kinases, triggers global reorganization of two critical hydrophobic “spines” within the kinase core that help to properly position key residues in the active site necessary for catalysis (Taylor and Kornev, 2011; Meharena et al., 2013). Of course, phosphorylation-dependent changes in activity are not restricted to enzymes. Indeed, many ion channels and cellular transporters are known to be regulated by phosphorylation. For instance, phosphorylation of ryanodine receptor (RyR) Ca^2+^ channels by either Ca^2+^/calmodulin-dependent protein kinase II (CaMKII) or cAMP-dependent protein kinase (PKA) induces conformational changes that alter their open probability at the sarcoplasmic reticulum, influencing both the intensity and the frequency of Ca^2+^ spikes in cardiomyocytes and striated muscle cells (Meissner, 2010; Niggli et al., 2013). While CaMKII-mediated phosphorylation of S2815 is believed to induce local conformational changes that directly affect the flow of Ca^2+^ ions through the channel, structural changes caused by phosphorylation of S2809 and/or S2030 by PKA appears to promote the dissociation of the stabilizing protein, calbastin 2, from a site far removed from the phosphorylation sites. These changes lead to “leaky” SR channels associated with aberrant contractile function (Meissner, 2010; Niggli et al., 2013).

In addition to modulating its activity, the phosphorylation status of a protein may also impact other cellular parameters important to its function, such as its subcellular location or its stability. For instance, hyper-phosphorylation of the regulatory domain of the transcription factor, nuclear factor of activated T cells 1 (NFAT1), promotes electrostatic interactions between negatively-charged phosphate groups and positively-charged Lys/Arg residues found within a conserved nuclear localization sequence (NLS) (Okamura et al., 2000). These interactions induce conformational changes in the regulatory region that effectively mask NFAT1's NLS, leading to a predominantly cytoplasmic distribution prior to dephosphorylation by the Ca^2+^/calmodulin-dependent protein phosphatase, calcineurin (CaN).

In the previous example, the phosphorylation status of NFAT1 determines whether its NLS is sterically blocked by the regulatory region or exposed to the cellular environment. This is important because the NLS is, itself, a site of interaction with importin family members involved in nucleocytoplasmic shuttling (Marfori et al., 2011). In fact, many phosphorylation-dependent conformational changes are believed to regulate protein–protein interactions. In some instances, such as for the NFAT1-importin and RyR-calbastin 2 interactions described above, the phosphate group(s) is not directly involved in mediating interactions with partner proteins. In contrast, many phosphorylation-dependent interactions rely upon direct recognition of phosphorylated residues by a diverse set of phosphoamino acid binding domains (PAABDs). For instance, many members of the SCF family of ubiquitin E3 ligases associate with their substrates via either Trp-Asp 40 (WD40) or leucine-rich repeat (LRR) PAABDs that specifically bind pSer/pThr residues located in phosphodegrons (Ho et al., 2006; Reinhardt and Yaffe, 2013). Phosphorylation-dependent interaction of SCF family members leads to ubiquitylation and subsequent degradation of the substrate by the 26S proteasome. In this way, phosphorylation serves as a key upstream signal which influences protein stability.

Over the past 20 years, many PAABDs, each exhibiting distinct substrate specificities and binding affinities, have been characterized. These include PAABDS that exclusively recognize either pTyr residues or pSer/pThr residues, as well as others that exhibit dual specificity, interacting with the phosphate monoester of all three hydoxyamino acids (Liao et al., 1999; Jin and Pawson, 2012; Reinhardt and Yaffe, 2013). While most pTyr binding domains fall within two large families: (1) the Src homology 2 (SH2) domain family (~120 distinct members) and (2) the phosphotyrosine binding (PTB) domain family (~27 distinct members) (Yaffe, 2002; Liu et al., 2006; Jin and Pawson, 2012), the diversity of pSer/pThr PAABD's is much greater. Indeed, as a testament to the prevalence of Ser/Thr phosphorylation in eukaryotes, 14 distinct families of PAABDs that recognize pSer/pThr residues have been described to date (Jin and Pawson, 2012). Such a large number of distinct PAABD families suggests that phosphorylation-dependent interactions are a widely employed means of regulating protein–protein interactions inside cells.

Together, phosphorylation-dependent changes in protein function, be it through modulation of enzymatic activity, changes in sub-cellular localization, alterations in protein stability, or regulation of protein–protein interactions (or a combination thereof), plays a major role in regulating cellular physiology. Therefore, it is important to understand what factors influence protein phosphorylation inside cells.

### Factors influencing protein phosphorylation inside cells

Inside the cell, the phosphorylation status of a particular phosphorylation site (phosphosite) is determined by the equilibrium between kinase-mediated phosphorylation, in the one direction, and phosphatase-mediated dephosphorylation in the other direction^1^. In order for a kinase to act on a cellular protein, several criteria must be met. For instance, a kinase and its substrate(s) must be expressed in the same cell and at the same time during development. This often involves several levels of control, including transcriptional regulation (e.g., control of gene expression), post-transcriptional regulation (e.g., regulation of mRNA stability) and modulation of protein levels (e.g., regulation of protein stability). Diverse and complementary experimental approaches have been developed to monitor these parameters, including reporter gene assays to track changes in transcriptional activation at a given promoter (Roura et al., 2013; van Rossum et al., 2013; Khan et al., 2014), quantitative real-time PCR (qPCR) and expression microarrays to measure the relative levels of mRNA transcripts (Skrzypski, 2008; Gorreta et al., 2012), and western blot analysis and fluorescent imaging techniques to measure changes in protein abundance over time (Wiechert et al., 2007; Chao et al., 2012). Though a comprehensive discussion of these techniques is beyond the scope of this review, the interested reader is referred to several recent reviews (Wilkins, 2009; van Rossum et al., 2013).

In addition to being co-expressed, a protein kinase and its substrate must physically interact with one another in order for phosphorylation to occur. Therefore, information about protein localization and protein–protein interactions is critical in determining whether a given kinase-substrate relationship (KSR) is likely to occur under physiological conditions. Traditionally, researchers have employed biochemical approaches to obtain this information. For instance, subcellular fractionation, generally achieved via differential centrifugation through a density gradient, has long been used to separate cellular organelles. When combined with western blot analysis, cellular fractionation is an effective means of determining the subcellular localization of a particular protein species. More recently, fluorescence imaging techniques, such as immunofluorescence and live cell imaging, have offered insights into the subcellular localization of proteins without disrupting the cellular architecture. Importantly, in the case of live cell imaging, dynamic changes in the subcellular distribution of a fluorescently tagged protein can be visualized in real-time within single cells. The advent of real-time super-resolution microscopy techniques based on photoswitchable fluorescent proteins (FPs) has dramatically increased the resolution afforded by live cell fluorescence imaging experiments (reaching ~20 nm resolution in living cells compared to >200 nm using standard fluorescence imaging) (Dedecker et al., 2012; Agrawal et al., 2013; Persson et al., 2013). While, in theory, this resolution permits the visualization of several structures important for cellular signaling, such as membrane microdomains (Honigmann et al., 2013) and large protein complexes (Biggs et al., 2011), it is still not sufficient to detect most protein–protein interactions directly. Instead, this information can be attained in several ways, as briefly summarized below.

Perhaps the most popular method for assaying protein–protein interactions is co-immunoprecipitation (co-IP). The immunoprecipitation step can be achieved using antibodies raised against either a specific protein-of-interest or an epitope tag, such as the hemagglutinin (HA)- or tandem affinity purification (TAP)-tags (Puig et al., 2001). Therefore, co-IPs can be used to detect interactions that occur in different cell types, ranging from naïve primary cells to transfected and/or genetically engineered cells. When coupled with quantitative tandem mass spectrometric (MS/MS) analysis (see Section “Top-Down” Methodologies: *In situ* Identification of Phosphosites), co-IP assays can yield a wealth of information about protein–protein interactions that occur under various cellular conditions. However, because many protein interactions occur in the context of multimeric protein complexes, in the absence of cross-linking agents, co-IPs do not provide definitive information about direct protein–protein interactions. Moreover, because the cells must be lysed prior to immunoprecipitation, weak and transient interactions are often missed using this approach.

In contrast, yeast two-hybrid analysis, which generally detects binary interactions, is able to detect both weak and strong interactions alike. However, it is important to note that, because both the bait and the prey proteins must be localized to the nucleus using this approach, interactions often occur within a cellular context much different from the one normally encountered by the proteins under study. Moreover, if the interaction is dependent upon post-translational modifications, such as phosphorylation or acetylation, the modification may not actually occur in yeast. In fact, this may be the case even if an ortholog of the appropriate modifying enzyme (e.g., the orthologous kinase or acetyl transferase) is present in yeast. Indeed, we and others have recently reported that many KSRs are not conserved between yeast and humans, despite the presence of orthologous kinase-substrate pairs (Mok et al., 2011; Hu et al., 2014).

Therefore, to directly visualize protein–protein interactions within the native cellular environment, several fluorescence imaging techniques have been developed (Ciruela, 2008; Shekhawat and Ghosh, 2011; Stynen et al., 2012). These include approaches based on fluorescence resonance energy transfer (FRET) between fluorescently tagged proteins (Padilla-Parra and Tramier, 2012; Zadran et al., 2012; Sun et al., 2013) as well as protein complementation assays (PCA), which rely on the interaction-dependent reassembly of N- and C-terminal fragments of FP color variants (Ciruela, 2008; Shekhawat and Ghosh, 2011). Not only do these techniques allow protein–protein interactions to be observed in many subcellular regions, but they also can be conducted in cell types (e.g., mammalian cells) that place the interacting proteins in the context of their endogenous regulatory networks.

Together, gene expression, subcellular localization and protein–protein interaction data can be used to construct extensive interaction networks that offer global information about the interactome under different cellular conditions (Pastrello et al., 2014). This information can be very useful in predicting KSRs that are likely to occur inside cells. For instance, protein–protein interactions, be they direct or indirect (e.g., those mediated by scaffold proteins), appear to be one of the strongest predicators of physiologically relevant KSRs (Newman et al., 2013). This is likely due to the key role that protein–protein interactions play in substrate selection, which is discussed below.

## Substrate selection: identifying the cellular targets of protein kinases and phosphatases

It is currently believed that ~40% of the proteins in the human proteome are phosphorylated at some point during their lifetime. By extension, since a given protein often contains multiple phosphorylation sites, the total number of phosphosites in the human proteome has been estimated to be ~100,000 sites (Zhang et al., 2002; Dephoure et al., 2013). Therefore, simply cataloging the complete complement of phosphosites in the human proteome, irrespective of their dynamic regulation or their functional consequences, is a seemingly daunting task. This task is made even more challenging by the fact that, in many cases, the phosphorylated form of a protein represents only a very small fraction of the total copies of that protein species inside the cell. That being said, while phosphosite identification is extremely important, simply knowing which sites are phosphorylated inside the cell only tells half of the story. Indeed, if we are to truly understand how dynamic phosphorylation networks are organized and regulated inside cells, it is important to know which kinases are actually mediating the phosphorylation event.

Therefore, to construct comprehensive maps of cellular phosphorylation networks, researchers have developed a series of experimental approaches that can be broadly grouped into two complementary categories, which we will refer to as “top-down” and “bottom-up” methodologies^2^. While “top-down” approaches begin at the cellular level and work their way down to the protein level, “bottom-up” approaches begin at the protein level and work their way up to the cellular level. Below, we describe each category and highlight how they can be used together to gain global insights into cellular phosphorylation networks.

### “Bottom-up” methodologies: *in vitro* analysis of kinase-substrate relationships

In “bottom-up” approaches, biochemical analysis of individual kinase-substrate pairs is carried out *in vitro* using purified protein components. Traditionally, phosphorylation is detected using either autoradiography or scintillation counting following incubation of the kinase-of-interest with a single substrate and radiolabeled ATP (e.g., [γ^32^P]-ATP or [γ^33^P]-ATP). In order to identify the site(s) of phosphorylation on the substrate, the initial screen is often followed by mutational analysis of putative phosphorylation sites. Due to safety concerns associated with radioactive assays and the high cost of radioisotope disposal, several non-radioactive assays have been developed based on alternative detection methods (Glickman, 2012). In many cases, these methods employ coupled enzymatic reactions that measure activity-dependent depletion of ATP. On the other hand, the development of generalizable phospho-detection reagents, such as highly sensitive anti-pTyr antibodies or biotinylated Phos-Tag phosphochelators (see discussion in Section “Top-Down” Methodologies: *In situ* Identification of Phosphosites below), allows direct visualization of phosphorylation on protein substrates using fluorescent or chemiluminescent detection methods (Kinoshita et al., 2004, 2012a).

Regardless of the detection method employed, detailed biochemical analysis can offer a wealth of information about individual KSRs, including the kinetics of the phosphorylation reaction, the site(s) of phosphorylation and, in some cases, the functional consequences of phosphate addition. Each of these parameters is important for understanding how phosphorylation-dependent signaling is achieved inside cells. However, due to the time that it takes to fully characterize a single KSR, traditional biochemical approaches are not well suited to high-throughput analysis. Therefore, to increase the rate of KSR discovery, researchers have developed phosphorylation assays based on functional protein microarrays (Ptacek et al., 2005; Mok et al., 2009; Popescu et al., 2009; Newman et al., 2013).

**Figure 1:**
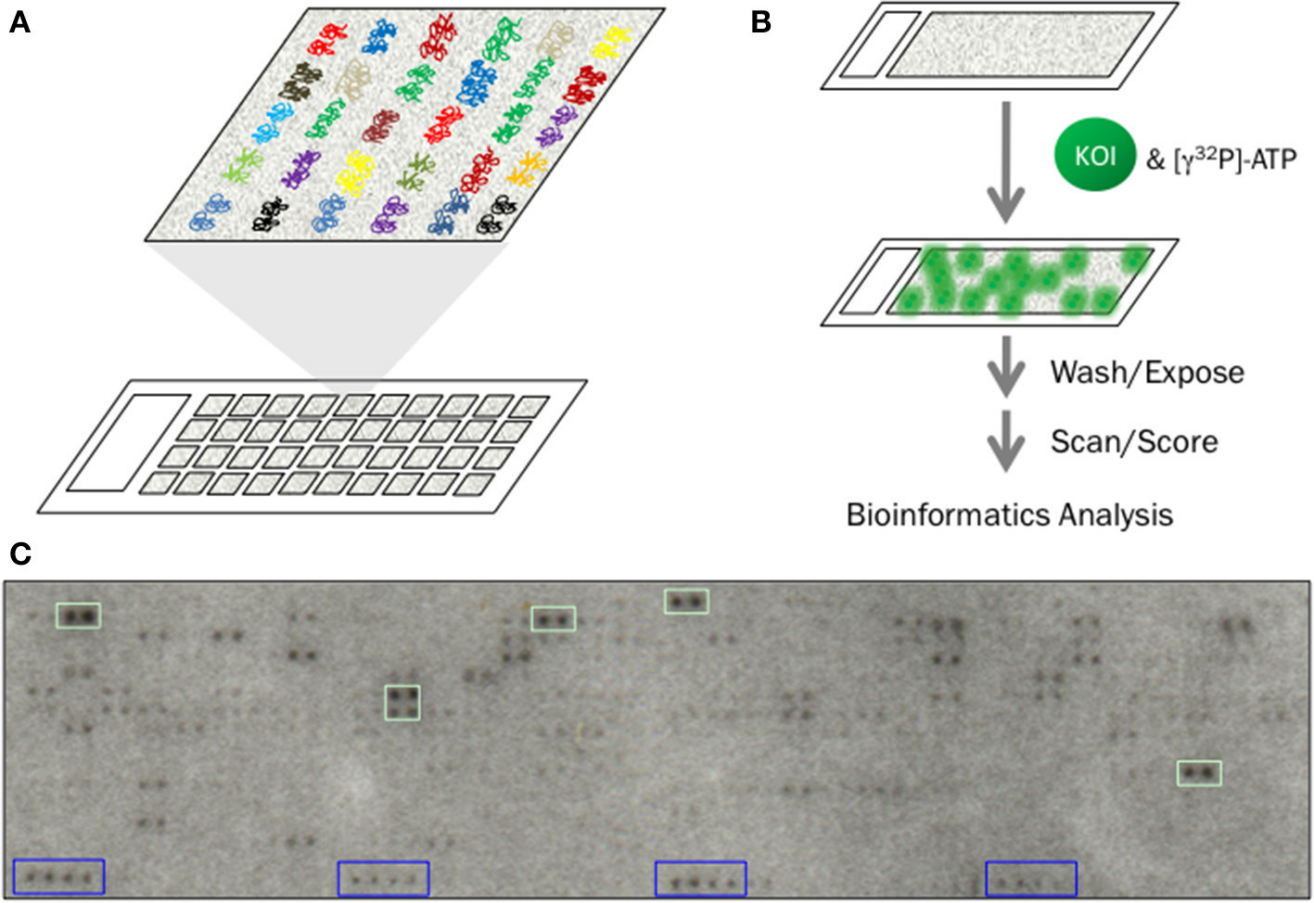
**Identification of KSRs using functional protein microarrays. (A)** Functional protein microarrays are composed of thousands of purified proteins immobilized on a functionalized glass surface. Each protein is printed in duplicate and in a defined location on the array. **(B)** Basic workflow for a phosphorylation assay using functional protein microarrays. Individual microarrays are first blocked and then incubated with the kinase-of-interest (KOI) in the presence of [γ^32^P]-ATP. Following incubation, the microarrays are washed extensively to remove unincorporated [γ^32^P]-ATP, dried and exposed to high-resolution X-ray film. Autoradiograms are then scanned and scored before being subjected to bioinformatics analysis to identify those KSRs that are likely to occur inside the cell. **(C)** Autoradiogram from a typical phosphorylation assay. Duplicate spots that each exhibit a normalized signal intensity 3 *SD* above the mean (green boxes) are considered positive hits. General kinase substrates, such as histone H3 and H4, printed in each block (blue boxes) are used as landmarks to orient the grid during scoring.

Functional protein microarrays are composed of thousands of individually purified recombinant proteins immobilized in discrete spatial locations on a functionalized glass surface (Hu et al., 2011; Sutandy et al., 2013). While several surface chemistries have been used to immobilize the purified proteins [e.g., polyvinylidene fluoride (PVDF), nitrocellulose, streptavidin and acrylamide] (Hu et al., 2011), bi-functional cross-linking agents, such as functionalized aminosilane and carboxylic esters, are generally preferable for phosphorylation assays due to their relatively low background signal (Mok et al., 2009). On the microarray, each individual protein is printed multiple times (typically in duplicate or triplicate) to ensure “on-chip” reproducibility and to guard against signal artifacts that can arise due to incomplete washing or experimental variation (Figure 1A). Because each spot on the array typically contains only a few femtograms of protein, sensitive detection methods are necessary to visualize phosphorylation. Moreover, since each protein occupies a defined position on the array and many proteins can potentially be phosphorylated in the same experiment, direct visualization of phosphorylation using either radioactive ATP or specific phospho-detection reagents (e.g., pTyr antibodies) is essential.

During a standard phosphorylation assay, each protein microarray is incubated with an active form of the kinase-of-interest in the presence of radioactive ATP (Figure 1B). Following incubation, the microarray is washed several times and dried by centrifugation before finally being exposed to high-resolution X-ray film for several hours or several days (Figure 1C). The resulting autoradiogram is then converted to a digital image and scored using image analysis software such as GenePix 6.0 (Axon, Inc.). General kinase substrates (e.g., histone H3 and H4) and/or kinases that undergo autophosphorylation are used as “landmarks” to align grids during scoring. In addition to the experimental arrays, it is important that each batch of experiments includes a negative control in order to identify those proteins that undergo autophosphorylation. Along with the landmarks, these species can also be useful for orienting the scoring grid during alignment. Using this protocol, a team of two researchers in our lab is able to conduct up to 144 phosphorylation assays per day, with each assay probing thousands of potential substrates. In this way, a large number of KSRs can be identified in a relatively short period of time. For instance, using high-density protein microarrays composed of 2158 unique proteins from *Arabidopsis thaliana*, Popescu and colleagues identified 570 substrates for 10 mitogen-activated protein kinase (MAPK) family members (Popescu et al., 2009). Likewise, we were able to identify over 24,000 *in vitro* substrates for 289 unique human kinases (Newman et al., 2013). While the latter studies used microarrays containing 4191 unique human proteins (Hu et al., 2009), the recent development of a high-density human protein microarray composed of 16,368 unique human proteins (representing over 80% of the human proteome) creates exciting new opportunities for KSR discovery at a global scale (Jeong et al., 2012).

Due to the large number of phosphorylated species generated during a typical microarray phosphorylation assay, sophisticated statistical analyses are often required to identify those substrates that constitute positive “hits.” First, the signal intensity of each protein spot is measured and subjected to some form of background correction, be it background subtraction or ratiometric analysis (e.g., calculation of the foreground/background ratio) (Ptacek et al., 2005; Popescu et al., 2009; Newman et al., 2013). To eliminate spatial artifacts that can arise due to uneven washing or drying of the microarray, local background correction is often preferable to global correction (Hu et al., 2009). If the normalized signal intensity for each of the replicate spots is above a predefined threshold [e.g., >3 standard deviations (SD) above the mean intensity on the microarray], then the protein is considered a putative substrate. However, it is important to note that, because both the kinase and the substrate are removed from the cellular environment, a positive result *in vitro* does not guarantee that the KSR actually occurs under physiological conditions. Bioinformatics analysis can help curb this limitation by identifying “high confidence” KSRs that are likely to occur inside the cell. Here, accurate gene ontology (GO) data, such as that obtained using the approaches outlined in Section Factors Influencing Protein Phosphorylation Inside Cells, is a valuable resource for determining whether a given cellular protein is likely to be phosphorylated by its cognate kinase *in vivo*.

Though necessary to eliminate false positives, the use of a stringent cutoff during KSR identification likely results in a relatively high false negative rate, as well (estimated to be ~95%, in some cases) (Newman et al., 2013). For instance, because the number of phosphosites for a given kinase varies from protein to protein, a substrate that contains only one phosphorylation site for a given kinase will exhibit a lower normalized signal intensity on the array than a protein that contains multiple sites for the same kinase. If the signal intensity of the first substrate falls below the threshold determined for the chip, then that substrate would not be considered a hit even though it is a *bona fide* substrate of the kinase (which likely would have been classified as such had the experiment been conducted using the purified kinase and substrate in isolation). Indeed, we have observed that known KSRs are sometimes absent from the final hit list. Visual inspection often reveals that this is not because the substrate is not actually phosphorylated on the microarray; rather it is because its normalized signal intensity does not exceed the stringent cut-off required to be considered a positive hit. Likewise, variations in protein abundance on the microarray or impairment of kinase-substrate interactions due to misfolding or truncation of the substrates during purification can bias KSR identification. Importantly, KSRs that are dependent on auxiliary factors, such as scaffolding proteins, co-factors, or post-translational modifications, may be missed all together using functional protein microarrays and other “bottom-up” approaches.

Therefore, as a complementary approach to the phosphorylation assays described above, protein microarray assays have been developed using cell lysates. For instance, Woodard et al. recently developed an assay platform that uses functional protein microarrays to measure phosphorylation profiles in whole-cell lysates obtained from cell or tissue samples (Woodard et al., 2013). This platform, which is similar to the phosphorylation assays described above except whole cell lysates are substituted for the purified kinase, revealed changes in the phosphorylation profiles of immortalized U373 cancer cells and gliablastoma tumors upon hepatocyte growth factor (HGF)-mediated activation of the c-Met signaling pathway. One of the primary advantages of this platform is that it more closely mirrors the cellular environment under which KSRs occur. For instance, because lysate assays preserve many of the elements of intact signaling networks, such as the endogenous levels of proteins, co-factors, second messengers, and inhibitors, while also maintaining protein complexes involved in signal transduction, they can offer insights into global changes in phosphorylation networks under different cellular conditions. Indeed, in addition to proteins known to be associated with the c-Met pathway, these analyses identified over 400 proteins that are likely influenced by HGF/c-Met signaling (Woodard et al., 2013). Moreover, the profiles obtained from these assays may be useful for biomarker discovery. Therefore, this approach has the potential to be a powerful diagnostic tool for rapid, cost-effective screening.

Similarly, reverse phase protein microarrays (RPPA) represent powerful tools for tracking changes in phosphorylation profiles across samples. Unlike the functional protein microarrays described above, RPPA fabrication entails the immobilization of cell lysates or tissue extracts on the functionalized glass surface. The arrays are then probed with an antibody specific for the epitope-of-interest (e.g., a particular phosphosite) to assess changes in its expression levels or phosphorylation status under different conditions. Because each spot contains only a few nanoliters of lysate, many arrays (each containing lysates derived from tens to thousands of unique samples) can be prepared using only a small amount of starting material (Pierobon et al., 2014). The microarrays can then be probed simultaneously, allowing a large number of analytes to be tested in parallel while reducing the effects of inter-assay variability. As a consequence, RPPA-based assays have been widely applied to the study of phosphorylation profiles in clinical samples. For instance, Ummanni et al. recently employed RPPA assays to probe the expression levels and phosphorylation status of 71 cancer-associated proteins in 84 non-small cell lung cancer cell lines (Ummanni et al., 2014). Interestingly, they found that the sensitivity toward the EGFR inhibitors, lapatinib and erlotinib, correlated more closely with EGFR/ERBB2 phosphorylation than with receptor expression levels. This was not the case for sensitivity toward the SRC/BCR-ABL inhibitor, dasatinib, which instead correlated with expression of proteins downstream of EGFR/ERBB2. Of course, a significant limitation of the RPPA approach is its dependence on the specificity and avidity of commercially produced antibodies. As the toolkit of reliable phosphospecific antibodies grows, so too will the utility of this approach.

It is important to note that, because many kinases are present in lysates simultaneously, the identity of the kinase actually mediating the phosphorylation of a given substrate cannot be definitively established using lysate-based approaches. Therefore, lysate-based assays should be considered orthogonal to assays using purified kinases. The same is true for other “top-down” approaches, as described below.

### “Top-down” methodologies: *in situ* identification of phosphosites

**Figure 2:**
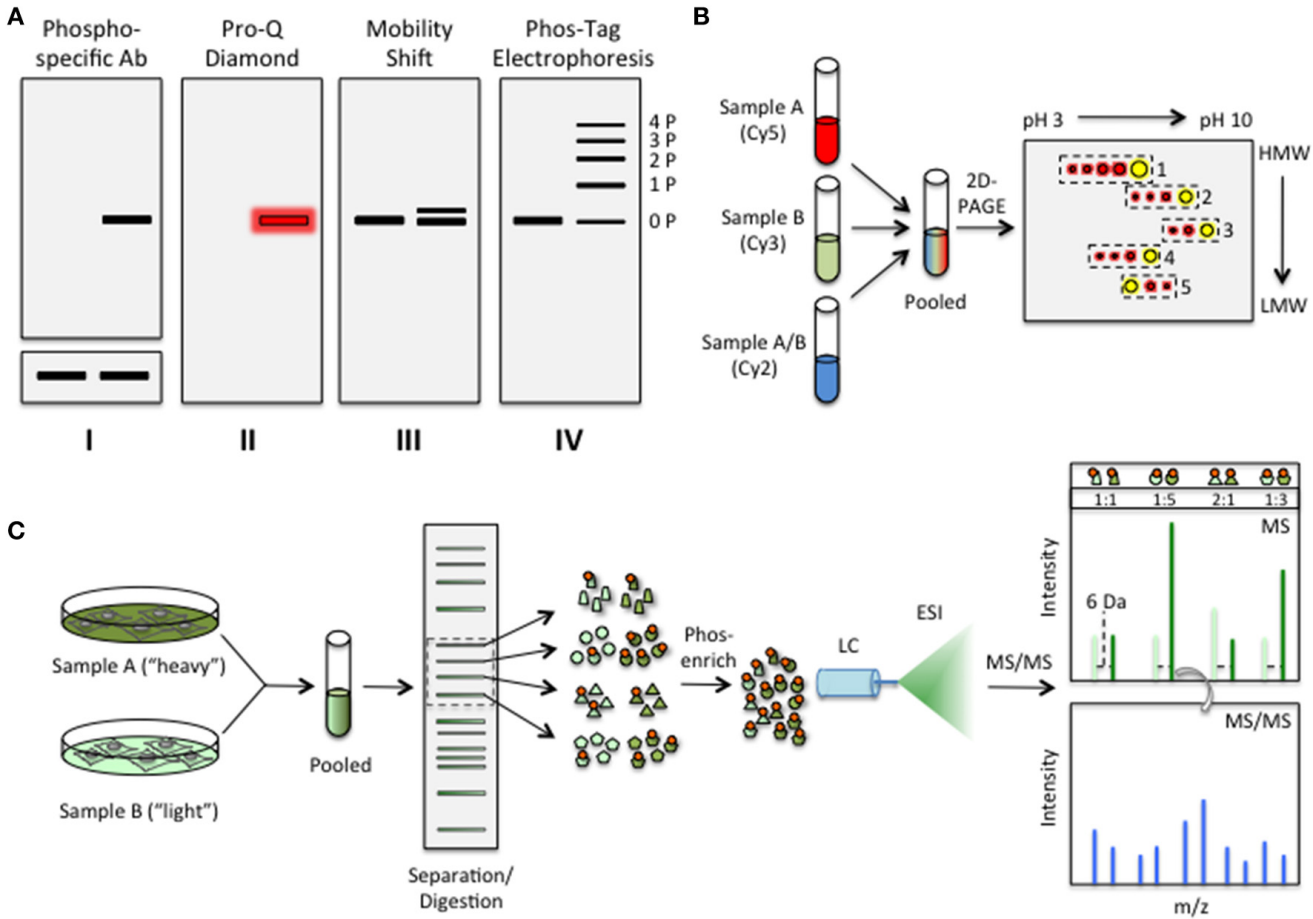
**“Top-down” approaches to assess changes in the phosphorylation status of cellular proteins. (A)** Approaches based on 1D PAGE. **I** Western blot analysis using an antibody that specifically recognizes a particular phosphosite. To account for phosphorylation-dependent changes in protein stability, the signal must be normalized to that obtained using an antibody that recognizes the unphosphorylated form of the protein-of-interest (below); **II** Detection using a general phosphorylation detection reagent, such as Pro-Q Diamond or the Phos-Tag phospho-chelator; **III** Phosphorylation of some proteins can be assessed based on changes in their electrophoretic mobility through a standard SDS-PAGE gel; **IV** Phos-Tag electrophoresis allows detection of a wide range of phosphorylation events, often resulting in a ladder corresponding to multiply phosphorylated species (1P, 2P, 3P, etc.). **(B)** Approaches based on 2D-PAGE. The general workflow of a 2D-DIGE experiment is shown. Accordingly, lysates from treated (Sample A) or untreated (Sample B) cells are first labeled with size- and charge-matched fluorescent dyes, such as Cy3 and Cy5, before being pooled together. A third sample, composed of both lysates labeled with a third dye (e.g., Cy2), may also be included as an internal reference. The pooled samples are then resolved on a 2D-PAGE gel, which generally uses IEF in the first dimension to separate cellular proteins based on their pI's followed by SDS-PAGE in the second dimension to separate the proteins based on size (HMW, high MW; LMW, low MW). Composite spots [e.g., Cy3 (green) and Cy5 (red)] are shown in yellow. Meanwhile, cellular proteins that are uniquely phosphorylated in the treated sample exhibit a spot train moving from right to left (e.g., boxes 1–4) while those proteins that are dephosphorylated under the experimental conditions are characterized by a spot train that moves in the opposite direction (e.g., box 5). The number of spots in the train corresponds to the number of phosphosites that are occupied in the protein (i.e., four spots represents four phosphorylation events). The intensity of each spot in the train can be used to gauge the relative levels of each phosphorylation state under each condition. **(C)** Approaches based on MS/MS. The basic workflow for a SILAC experiment is shown. To metabolically label proteins, cells are grown in the presence of either a “heavy” isotope of a particular amino acid (e.g., ^13^C-Arg) or its “light” counterpart (e.g., ^12^C-Arg). Cells are then pooled and lysed. Cellular proteins are resolved by 1D SDS-PAGE before being digested in the gel by an Arg/Lys-directed protease, such as trypsin. Peptide fragments in individual gel slices (represented by either parallelograms, circles, triangles, or pentagons) are then electro-eluted and phosphorylated species are enriched using one of several phospho-enrichment strategies outlined in the text. Phosphopeptides are then resolved by reverse-phase liquid chromatography (LC) and ionized by electrospray ionization (ESI) before being analyzed by in-line MS/MS. In the MS spectrum, fragments containing heavy isotopes are off-set by a known amount (e.g., 6 Da for ^13^C-Arg), allowing quantitation based on the relative intensity of each peak. The identity of individual peaks is determined based on the MS/MS spectrum.

“Top-down” approaches to substrate identification are designed to measure changes in the phosphorylation status of cellular proteins upon activation/inhibition of endogenous signaling pathways. Traditionally, this has been achieved using polyacrylamide gel electrophoresis (PAGE)-based methods (Figures 2A,B). Following cell lysis, the protein-of-interest is either immunoprecipitated or the whole cell lysate is loaded directly onto the gel. Separation is generally achieved via either one- or two-dimensional PAGE. In the case of one-dimensional PAGE, electrophoresis through a denaturing gel is often followed by western blot analysis to visualize the phosphorylation status and/or the migration pattern of the protein-of-interest (Figure 2A). If a phosphospecific antibody exists for the phosphosite under study, changes in phosphorylation levels at that site can be assessed directly by comparing the intensities of the bands before and after treatment (Figure 2AI). However, because phosphorylation can impact protein stability inside the cell, the signal intensities obtained using phosphospecific antibodies must be normalized to the amount of target protein in each lane (e.g., by using an antibody raised against the unphosphorylated form of the protein). If a phosphospecific antibody does not exist (as is often the case) or the specific site of phosphorylation is not known, the phosphorylation status of proteins may still be assayed using general phospho-detection reagents, such as Pro-Q Diamond (Life Technologies, Inc.) or the Phos-Tag phosphochelator, or based on changes in the electrophoretic mobility of the protein (Figures 2AII,III). In the case of the latter, it is believed that the high charge density of the phosphate monoester prevents the uniform binding of negatively charged SDS molecules in the vicinity of the phosphate group, causing some phosphorylated proteins to migrate more slowly than their unphosphorylated counterparts (Gafken and Lampe, 2006). For instance, using a library of epitope-tagged proteins, the Matsuda group profiled changes in the electrophoretic mobility of each of the proteins in the *S. pombe* proteome (Shirai et al., 2008). While this study did not focus specifically on phosphorylation, the authors noted that nearly 42% of the proteins in the *S. pombe* proteome migrated at a molecular weight that was significantly different from their calculated molecular weight (with ~28% of the proteins migrating at a higher-than-expected MW).

However, it is important to note that not all proteins exhibit an observable mobility shift upon phosphorylation. Therefore, to accentuate phosphorylation-induced changes in mobility in an unbiased manner, Kinoshita and colleagues recently developed several methodologies based on so-called Phos-Tag technologies (Kinoshita et al., 2004, 2012a,b; Tsunehiro et al., 2013). Phos-Tags, which are alkoxide-bridged dinuclear metal complexes, have been shown to selectively bind phosphate monoesters in a sequence-independent manner (Kinoshita et al., 2004). When conjugated to a gel matrix such as acrylamide, the Phos-Tag effectively slows the migration of any species containing a phosphate monoester, leading to a discernable shift in its mobility (Kinoshita et al., 2012b) (Figure 2AIV). Consistent with the notion that many proteins are multiply phosphorylated inside the cell, a ladder of clearly distinguishable bands is often observed using Phos-Tag electrophoresis. Though several studies have used Phos-Tag technologies either to profile the phosphorylation status of select substrates (Kinoshita et al., 2009; Aguilar et al., 2011; Kinoshita-Kikuta et al., 2012) or to validate substrates identified by other methods (Mukai et al., 2008; Deswal et al., 2010; Mok et al., 2010; Huang et al., 2014; Yip et al., 2014), to date Phos-Tag electrophoresis has not been applied to large-scale proteomic analyses. It will be interesting to compare the results of such studies with those obtained using standard 1D SDS-PAGE—such as the one conducted by Matsuda et al. in *S. pombe* (Shirai et al., 2008)—as well as with those that utilize 2D-PAGE, as described below.

In addition to one-dimensional PAGE, 2D-PAGE is can also be used to assess the phosphorylation status of cellular proteins. In a 2D-PAGE experiment, proteins are typically separated via isoelectric focusing (IEF) in the first dimension followed by SDS-PAGE in the second dimension (though other separation strategies have also been successfully combined) (Camacho-Carvajal et al., 2004; Kinoshita et al., 2009) (Figure 2B). Because the negative charge of the phosphate monoester causes a reduction in the pI of the protein, the migration of the phosphorylated species is altered during IEF. Separation in the second dimension therefore leads to clearly discernable spot trains along the vertical or horizontal axes, corresponding to differentially phosphorylated species. When coupled with western blot analysis, 2D-PAGE can offer insights into either the phosphorylation status of a specific cellular protein or global changes in phosphorylation across the entire proteome (e.g., changes in Tyr phosphorylation). Alternatively, proteins can first be stained with a contrast agent, such as Coomassie dye or silver stain, then excised from the gel, and sequenced using either Edman sequencing or quantitative MS/MS.

One of the greatest strengths of 2D-PAGE is that it simultaneously offers insights into the relative expression levels and post-translational modifications of intact cellular proteins. At the same time, this also highlights one of its limitations with respect to phosphosite identification. Indeed, because phosphorylated proteins are often present at relatively low levels inside cells, many phosphoproteins can be missed using this approach. In some cases, this limitation can be overcome through the use of sensitive phosphodetection reagents, such as radioactive ATP or phosphospecific antibodies, or through phosphoprotein enrichment procedures, such as immobilized metal affinity chromatography (IMAC) using trivalent metal ions such as Fe^3+^ and Ga^3+^ (Posewitz and Tempst, 1999; Kosako and Nagano, 2011). Likewise, subcellular fractionation can be used to examine the phosphorylation profiles of proteins within specific subcellular compartments (Stasyk et al., 2007).

To gain insights into differential phosphophorylation of cellular proteins using 2D-PAGE, two sets of lysates must be compared (e.g., cell extracts from healthy vs. diseased cells). However, due to inherent technical obstacles stemming from gel-to-gel variability and differential staining of proteins, cross-gel comparisons are often difficult using this approach. To address these limitations, researchers have developed 2D-differential gel electrophoresis (2D-DIGE) methodologies (Minden, 2012) (Figure 2B). In a 2D-DIGE experiment, spectrally distinct, charge-matched fluorescent dyes, such as Cy3 and Cy5, are used to label the proteins in individual lysates. The two lysates, each labeled with a distinct dye, are then mixed and examined by 2D-PAGE. Because 2D-DIGE permits the analysis of multiple lysates on a single gel, direct comparison between different experimental conditions is relatively straightforward. Moreover, due to their high intrinsic brightness, the fluorescent dyes used for labeling are well-suited for the detection of low abundance protein species (Mujumdar et al., 1993; Kosako and Nagano, 2011). Importantly, quantitation of the fluorescence intensity of each spot also allows relative levels of phosphorylation to be measured directly. Using this approach, Pellegrin et al. conducted a proteome-wide analysis of primary erythrocytes undergoing apoptosis following erythropoietin withdrawal (Pellegrin et al., 2012). These studies revealed changes in both protein abundance and the phosphorylation profiles of many important cellular proteins, including several heat shock protein 90 (Hsp90) isoforms.

Despite the improved sensitivity and analytical power afforded by 2D-DIGE, methods based on 2D-PAGE are still only able to identify changes in a few hundred proteins at a time (Choudhary and Mann, 2010). This makes it difficult to gain a truly systems-level view of phosphorylation networks, where thousands of different cellular proteins may be phosphorylated at any given time. In this respect, the development of shotgun proteomics approaches based on MS/MS has been truly revolutionary. Indeed, studies employing shotgun MS/MS have recently led to an explosion in the number of annotated cellular phosphorylation sites, opening new avenues of research in systems biology.

Like the other “top-down” approaches discussed above, MS-based detection of phosphosites requires cell lysis prior to analysis. Following lysis, proteins are generally resolved by a separation technique suitable to the sample volume to be analyzed (e.g., SDS-PAGE or column chromatography) and digested using an Arg/Lys-directed protease, such as trypsin, chymotrypsin or lysyl endopeptidase (Figure 2C). Phosphopeptides are then enriched using one of several phosphoenrichment procedures discussed below. The enriched phosphopeptides are separated by reverse-phase liquid chromatography (LC) before being ionized by electrospray ionization as they elute from the column. Finally, the ionized peptides are loaded onto an in-line mass analyzer for analysis. In the case of MS/MS, two mass analyzers, for example an ion trap instrument and an orbitrap instrument, are often used in tandem to increase the resolution of detection^3^. In this scheme, the first mass analyzer is used to obtain a mass spectrum of all peptides eluting from the column at a given time. Once the first mass spectrum is obtained, peptides with a specific mass-to-charge ratio (m/z) are isolated, further fragmented through high-energy interactions, and analyzed by the second mass analyzer (modern mass spectrometers can isolate individual peptides in a matter of milliseconds) (Choudhary and Mann, 2010). The tandem mass spectra obtained from these experiments are then compared against protein sequence databases or *in silico* peptide fragmentation spectra in order to match the peptide fragments to a corresponding cellular protein (Gafken and Lampe, 2006). A 79.97 Da increase in the mass spectrum of a peptide fragment is indicative of phosphorylation; however, it is important to note that, if multiple Ser/Thr/Tyr residues are present in close proximity to one another in a peptide, it is often difficult to unambiguously identify the site of phosphorylation.

Though MS analysis has been used for decades to analyze relatively simple mixtures consisting of a few hundred proteins, recent advances have now made possible the identification of thousands of phosphopeptides in a single study. As a consequence, MS-based studies have contributed to an unprecedented increase in the number of known phosphoproteins and phosphosites, which currently stands at just under 118,500 unique phosphosites in ~16,400 non-redundant proteins across human, mouse, and several other species [PhosphositePlus database (as of 4/13/2014)]. As we discuss below, this veritable explosion in phosphosite data has been made possible by several key technological advances in MS-based analysis of the phosphoproteome, including (1) the development of phosphopeptide enrichment methods, (2) improvements in the resolution and accuracy of mass analyzers, (3) the emergence of alternative fragmentation methods, and (4) the development of computational algorithms that allow high-confidence peptide identification and phosphosite localization. Moreover, the introduction of quantitation strategies based on isotopic labeling allows researchers to track dynamic changes in global phosphorylation profiles in response to a particular cellular stimulus. Due to the existence of a number of excellent reviews on these topics, (Choudhary and Mann, 2010; Kosako and Nagano, 2011; Johnson and White, 2012; Kinoshita-Kikuta et al., 2012; Nilsson, 2012; Ong, 2012; Rigbolt and Blagoev, 2012) here we will only highlight these innovations and briefly discuss how they have been used to gain a systems-level view of phosphorylation networks.

As alluded to above, the occupancy of many phosphosites is sub-stoichiometric. As a consequence, at any given time, only a small fraction of the total copies of a protein species are expected to be phosphorylated at a particular site. When coupled with the fact that many phosphoproteins involved in signal transduction are already expressed at relatively low levels, detection of phosphorylated species represents a major challenge to phosphosite identification. To overcome these challenges, researchers have recently developed a variety of phoshpoenrichment techniques designed to selectively bind phosphorylated peptides. In addition to the IMAC- (Andersson and Porath, 1986; Posewitz and Tempst, 1999) and Phos-Tag-based (Nabetani et al., 2009) approaches discussed above, phosphoenrichment strategies using chromatographic separation by strong cation exchange (SCX), hydrophilic interactions, or immobilized metal oxides, such as titanium dioxide (TiO_2_) and ziroconium dioxide (ZrO_2_), have also been successfully combined with MS/MS for phosphosite identification (Ruprecht and Lemeer, 2014; Yang et al., 2014). Likewise, immunopurification using phosphospecific antibodies, such as anti-pTyr antibodies or antibodies raised against the phosphorylated form of the consensus phosphorylation motif of a kinase-of-interest (Zhang et al., 2002), provide information about phosphorylation on a particular type of residue (e.g., Tyr) or in a particular sequence context (e.g., a kinase consensus motif), respectively. Due to their high degree of enrichment and ease of use, IMAC- and metal oxide-mediated enrichment strategies are currently the most popular methods for phosphoproteomic analysis. However, these methods are also prone to non-specific interactions with acidic peptides containing a disproportionate number of Asp and Glu residues. Therefore, it is sometimes necessary to combine one or more enrichment methods prior to analysis by LC-MS/MS.

In addition to the development of phosphoenrichment methods, improvements in the resolution and mass accuracy of modern mass analyzers have greatly improved our ability to detect phosphosites in complex mixtures. For instance, whereas standard ion trap mass spectrometers can typically resolve a few hundred to a thousand individual peptides as they emerge from the reverse phase column, modern time-of-flight (TOF) and orbitrap instruments exhibit a mass resolution of 20,000 and 100,000, respectively. Moreover, these instruments exhibit mass accuracies in the low parts-per-million, dramatically improving the coverage that can be achieved in a single experiment. Together, the increased resolution and mass accuracy afforded by modern mass spectrometers allows much more complex mixtures to be analyzed at once, markedly improving both the throughput of a given experiment and the confidence in the resulting peptide assignments.

One of the primary challenges in phosphosite detection is preservation of phosphorylation during peptide fragmentation. This is because collision-induced dissociation (CID) methods, which accelerate the ionized peptides in a collision cell and then bombard them with inert gases such as helium, nitrogen, or argon, tend to cause the cleavage of the phosphate monoester linkage on hydroxyamino acid residues (Kosako and Nagano, 2011). The development of “phosphate-friendly” fragmentation methods, such as higher energy collisional dissociation (HCD) (Olsen et al., 2007), electron capture dissociation (ECD) (Zubarev et al., 2000), and electron transfer dissociation (ETD) (Syka et al., 2004), permits efficient fragmentation without cleavage of the phosphoester bond. As a consequence, more phosphosites will be preserved and available for detection. Importantly, because the fragmentation mechanisms used by ETD and CID are complementary, these methods can be used together to improve identification of phosphopeptides (Na and Paek, 2014).

Once mass spectra are generated, peptide sequences are typically identified by comparing the spectra with databases specific for the species under study. A number of currently available database search algorithms, such as MASCOT, SEQUEST, X!Tandem, and the Open Mass Spectrometry Search Algorithm (OMSSA), (Eng et al., 1994; Perkins et al., 1999; Craig and Beavis, 2003; Geer et al., 2004) are amenable to the identification of phosphorylated peptides. In general, these algorithms compare the experimentally determined mass spectra to theoretical fragmentation spectra generated *in silico*. Various scoring metrics are then used to determine the likelihood that a given match represents a *bona fide* phosphorylation event.

In addition to database searches, *de novo* sequencing approaches have also been developed. Unlike database searches, which are restricted by available sequence data, modern *de novo* approaches, such as DeNovoPTM, (He et al., 2013) infer the amino acid sequence (and any modifications thereto) directly from the MS/MS spectrum. Consequently, they are able to detect modifications in mutated proteins that may otherwise be missed. However, because *de novo* approaches are highly dependent on the quality of the spectral data, they are often prone to errors caused by incomplete or random fragmentation patterns (Na and Paek, 2014). To overcome these limitations, hybrid methods have been developed that conduct the database search using short sequence tags composed of two to four amino acids (determined by *de novo* sequencing). The use of sequence tags, which are less sensitive to sequencing errors than complete *de novo* sequencing, can dramatically reduce the database search time by reducing the search space to only a few candidate peptides that contain the sequence tag. By comparing the experimentally determined mass spectra with the theoretical spectra associated with the sequence tag, hybrid approaches have the potential to rapidly identify phosphorylation events on peptides (as well as many other modifications).

However, as alluded to above, in many cases a phosphorylated peptide may contain multiple phosphorylatable residues. In these cases, the unambiguous assignment of phosphosites is a non-trivial task. Therefore, to facilitate phosphosite identification, programs such as MASCOT and MSQuant include algorithms that assign a confidence score to each Ser, Thr, and Tyr residue present in a peptide sequence. Similar algorithms, such as the Ascore algorithm, can also be applied to MS spectra to facilitate phosphosite identification (Beausoleil et al., 2006; Olsen et al., 2006; Savitski et al., 2011).

Finally, due to the complex nature of biological samples and the run-to-run variations that can occur at several steps in the fractionation/detection protocol, traditionally it has been difficult to monitor changes in the phosphorylation status of cellular proteins using MS/MS-based approaches. However, the development of quantitative MS methods, such as stable isotope labeling of amino acids in cell culture (SILAC) (Ong, 2012) and isobaric tags for relative and absolute quantitation (iTRAQ) (Evans et al., 2012), have made it possible to directly compare phosphorylation profiles of multiple samples in a single experiment. These approaches, which rely on isotopic labeling of protein and peptide fragments, respectively, have quickly become mainstays in systems biology research.

In SILAC, two sets of cells are grown in culture media containing either a stable isotope of a particular “heavy” amino acid(s) (usually ^13^C-Arg and/or ^13^C-Lys) or its normal, “light” counterpart (Figure 2C). As a consequence, cellular proteins are metabolically labeled with either the heavy or the light isotope. Following lysis, the differentially labeled lysates are mixed together and subjected to identical digestion, separation, enrichment, and detection procedures. Meanwhile, iTRAQ and its close cousin, tandem mass tag (TMT) (Dayon and Sanchez, 2012; Jia et al., 2012), incorporate an isotopically labeled tag on peptide fragments *in vitro* following protease digestion. Because peptide fragments are labeled after lysis using iTRAQ and TMT, these approaches are amenable to analysis of primary cells as well as cultured cells. However, as with any *in vitro* reaction, care must be taken to minimize side reactions that can obscure analysis.

The power of isotopic labeling techniques stems from the fact that isotopic isomers are chemically identical—and therefore behave identically during LC-MS/MS—but can be distinguished from one another during detection due to a measurable offset between their m/z ratios (Figure 2C). As a consequence, relative changes in the phosphorylation status at a particular site can be measured by calculating the ratio of intensities between the heavy and light isotopes for each fragment. These quantitative MS approaches have proven to be powerful means of tracking system-wide changes in phosphorylation networks in a variety of cellular contexts and, as we will see in Section Computational Models of Phosphorylation Networks, provide critical information that facilitates systems-level modeling of dynamic phosphorylation networks.

Together, the advances in MS technology described above have led to the identification and, in many cases quantitative profiling, of a very large number of phosphosites that occur within the cell. Indeed, the identification of thousands, and even tens of thousands, of phosphosites in a single study is often achieved (Choudhary and Mann, 2010). For instance, in a SILAC-based experiment, Olsen et al. employed SCX/TiO_2_ enrichment and ion trap-orbitrap MS/MS to identify over 20,400 unique phosphorylation sites on ~6000 cellular proteins isolated from HeLa cells undergoing mitosis (Olsen et al., 2010). In addition to recovering a large percentage of known phosphosites involved in the mitotic transition, this study also uncovered nearly 10,000 previously unknown sites. More recently, Mertins et al. used an i-TRAQ-based protocol to profile ischemia-induced changes in the phosphoproteomes of human ovarian tumor and breast cancer xenograft tissues (Mertins et al., 2014). Among the >25,000 phosphosites identified in these studies, approximately one quarter of them (24%) were either up- or down-regulated in response to short periods of ischemia (<60 min). The altered phosphosites were predominantly associated with stress-response pathways, such as EGFR and MAPK pathways, suggesting that these pathways are activated at very early stages during the ischemic response.

Together, large-scale MS-based studies have greatly expanded the number of annotated phosphoproteins and phosphosites in the phosphoproteome. These data have facilitated global analysis of phosphorylation networks and provided important insights into the cellular targets of protein kinases and phosphatases. However, as alluded to above, MS/MS and other “top-down” approaches are unable to definitively match a given phosphorylation event with the upstream kinase or phosphatase. This notion is underscored by several recent studies that directly examined the interconnectedness of components within phosphorylation-dependent signaling networks. For instance, Bodenmiller et al. employed targeted gene disruption and select analog sensitive kinase (as-kinase) variants to conduct a systems-wide analysis of yeast phosphorylation networks (Bodenmiller et al., 2010). Using quantitative MS/MS, they identified over 8800 phosphorylation events that are regulated by 97 kinases and 27 phosphatases. Not surprisingly, in addition to direct KSRs, they also observed phosphorylation events that were modulated indirectly by disruption of the target kinase/phosphatase activity. Perhaps more interestingly, these analyses revealed that the number of indirect interactions greatly outnumbered the number of direct interactions, accounting for ~2/3 of the total regulated phosphorylation events in the case of kinase gene disruption. Similarly, Breitkreutz et al. generated an extensive kinase-phosphatase interaction (KPI) network in yeast and used it to examine the relationships between phosphorylation-dependent signaling modules. These studies revealed locally dense regions within the network centered around several key signaling molecules, such as the Ser/Thr phosphatase, cell division cycle 14 (Cdc14), and the Ser/Thr kinases, target of rapamycin complex 1 and 2 (TORC1 and TORC2) (Breitkreutz et al., 2010). Within this network, they also uncovered a significantly enriched kinase–kinase (K–K) interaction network that was extremely robust to fragmentation by hub deletion. A similar K–K network is highly conserved between yeast and humans (Newman et al., 2013). Together, these studies suggest that components within phosphorylation-dependent signaling networks are highly interconnected, exhibiting far less modularity than would be expected from the simple linear pathways typically used to depict cellular signaling pathways. As a consequence, modulation of a single network component (e.g., an upstream kinase) is likely to impact the phosphorylation status not only of its immediate downstream substrates, but also that of a large number of substrates that are not directly connected to the target within the network. This makes it extremely difficult to identify direct KSRs for the majority of kinases *in vivo*. Therefore, in addition to the identification of additional phosphoproteins and phosphosites, a major challenge in systems biology research has been to match annotated phosphosites to their upstream kinases and/or phosphatases. This requires information about phosphosites that occur under various cellular conditions, derived from “top-down” approaches, as well as information about direct KSRs, obtained by “bottom-up” approaches. To this end, several methods have been developed to integrate data obtained *in vitro* and *in vivo* to create high-resolution maps of phosphorylation networks.

### Combined approaches to connect phosphosites to their cognate kinases

**Figure 3:**
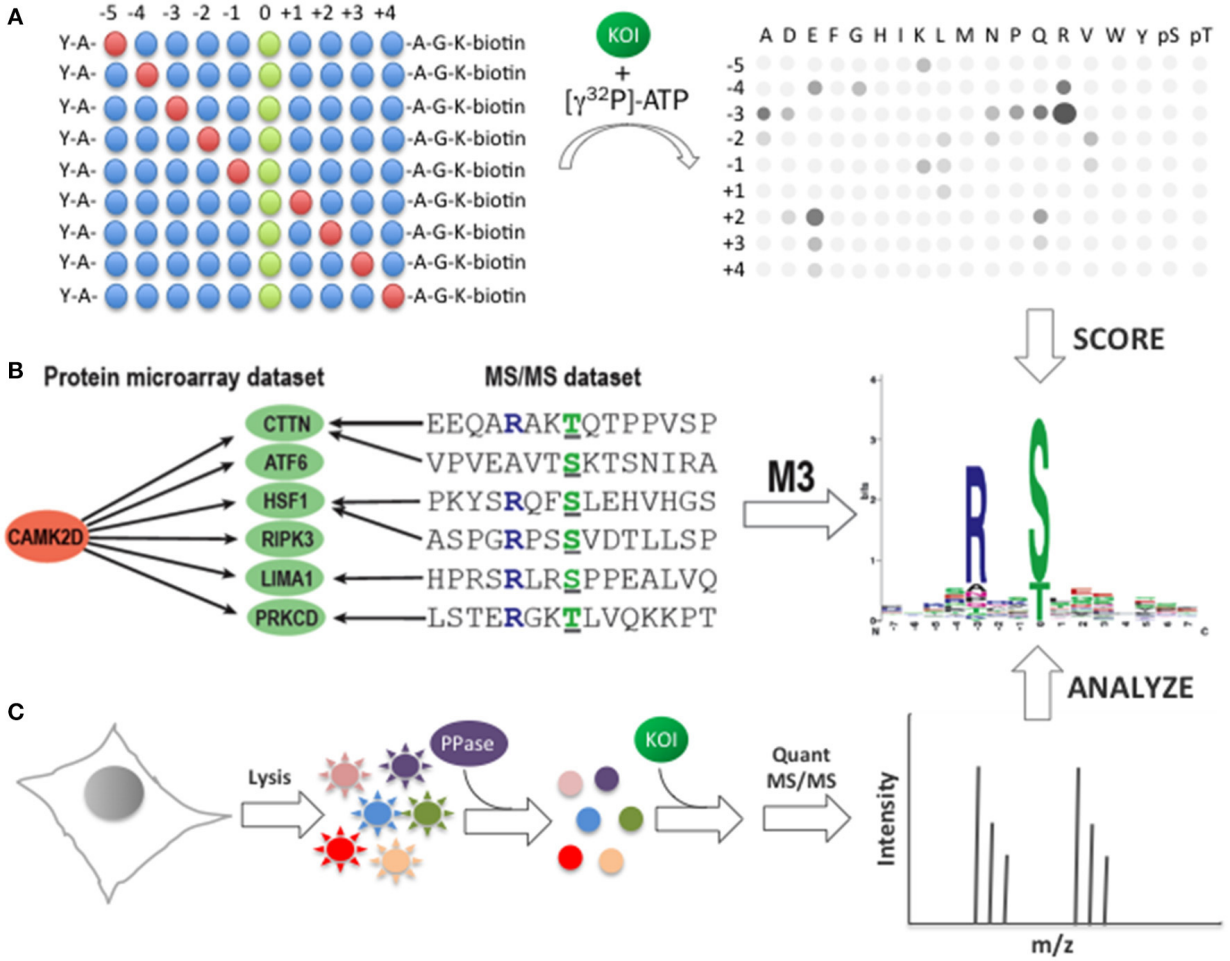
**Identification of consensus phosphorylation motifs. (A)** Determination of phosphorylation motifs using scanning peptide arrays. A library of biotinylated peptides is first synthesized in which one position is fixed (red circles) at a defined position relative to the phosphoacceptor site (green circles). The fixed position can be any of the 20 canonical amino acids, as well as pThr or pTyr. All other positions in the peptide mixture contain equimolar amounts of the canonical amino acids, excluding Ser, Thr, and Cys (blue circles). To determine the consensus motif, each peptide mixture is incubated with the kinase-of-interest (KOI) in the presence of [γ^32^P]-ATP before being immobilized on a streptavidin-coated membrane (right). The membrane is then washed, dried, and imaged. The resulting autoradiogram can be used to determine a consensus phosphorylation motif for the KOI based on the relative intensity of each spot in the array. **(B)** Consensus phosphorylation motif identification using the M3 algorithm. Known *in vivo* phosphorylation sites, determined primarily by MS/MS analysis, are first mapped onto each of the substrates of the KOI (e.g., CAMK2D) identified by phosphorylation assays using functional protein microarrays. M3 then uses an iterative approach to identify those residues that are enriched at each position relative to the phosphoacceptor site. The resulting matrix is used to construct a consensus phosphorylation motif for the KOI. **(C)** Consensus phosphorylation motif prediction using quantitative MS/MS. Cell lysates are first dephosphorylated, then incubated with the KOI and analyzed by quantitative MS/MS using phospho-enrichment. Statistically-overrepresented residues are used to construct a consensus phosphorylation motif for the KOI.

Methods designed to connect phosphosites to their immediate upstream kinase(s) generally require information about consensus phosphorylation motifs (Figure 3). Traditionally, consensus phosphorylation sites have been derived from positional scanning peptide arrays (Figure 3A). For instance, positional scanning peptide arrays have been designed to identify phosphorylation motifs for Ser/Thr kinases (Chen and Turk, 2010). These arrays are composed of a collection of synthetic peptides containing a central phosphoacceptor site surrounded by degenerate positions containing equimolar amounts of 17 amino acids (Cys, Ser, and Thr are generally excluded). In each peptide, one position (encoding either one of the 20 common amino acids or pThr or pTyr) remains fixed while all other positions are varied. Each peptide mixture is then incubated with the kinase-of-interest in the presence of radioactive ATP, immobilized on a functionalized glass surface, and washed extensively. In a related approach using so-called peptide SPOT microarrays, the peptides are immobilized on a functionalized glass surface prior to incubation with the kinase-of-interest (Leung et al., 2009). After washing, the intensity of each spot is used to identify those residues that are preferred by the kinase-of-interest at a particular position relative to the phosphoacceptor site. Using this approach, consensus phosphorylation motifs have been determined for a number of important protein kinases, including PKA, Src, Akt, PIM1, and PKC.

One of the primary advantages of peptide arrays for consensus phosphorylation motif discovery is that the peptides in the matrix are relatively well defined (at least to the extent that the phosphoacceptor site and the fixed position are constant and all other positions contain equimolar amounts of the other amino acids). However, because the length of the derived motif is limited by the number of residues in the peptides and post-translational modifications other than pTyr or pThr are not present in the synthetic peptides, this approach may lack important information regarding the molecular determinants of kinase specificity. Moreover, since the peptides are not in the context of the full-length protein substrate, other factors involved in substrate recognition, such as docking sites or tertiary structure, are absent. Therefore, as a complementary approach, we developed an algorithm, termed Motif discovery based on protein Microarrays and MS/MS (M3), that combines data about KSRs determined *in vitro* using functional protein microarrays with information about phosphorylation sites identified *in vivo* by MS/MS analyses (Newman et al., 2013) (Figure 3B). M3 first maps *in vivo* phosphorylation sites onto the each of the substrates identified on the microarray for a given kinase. It then uses an iterative approach to identify those residues that are enriched at various positions around the phosphoacceptor site. Using this approach, we predicted consensus motifs for each of the 289 unique kinases in our human kinase collection (including distinct Tyr and Ser/Thr motifs for several dual specificity kinases). When motifs generated by M3 were compared to those generated by positional scanning peptide arrays, a high degree of similarity was observed between them.

One of the greatest strengths of M3 is its flexibility. For instance, though we chose to examine 15-mers in our study in order to facilitate comparison with motifs determined using scanning peptide arrays, in theory, motifs of various lengths can be generated in a relatively straightforward manner. Likewise, because M3 includes information about the entire amino acid sequence surrounding the putative phosphoacceptor site, aside from information about which residues are preferred at a particular position, it also provides insights into which residues are *disfavored* at select positions. This information may be particularly useful in understanding the substrate selectivity of kinases that have very similar motifs (e.g., closely-related members of the same kinase family). That being said, because it uses an iterative approach for motif discovery, M3 requires a relatively large number of substrates/phosphosites for accurate motif prediction. Likewise, the accuracy of M3 is limited by the number of known KSRs, on the one hand, and the number of annotated *in vivo* phosphosites, on the other. As these data sets continue to expand, it is likely that the accuracy of M3 with continue to improve, as well.

In addition to the microarray-based strategies described above, several groups have recently developed MS/MS-based approaches for motif discovery (Huang et al., 2007; Chou et al., 2012; Kettenbach et al., 2012; Knight et al., 2012) (Figure 3C). In general, these methods rely on the phosphorylation of eukaryotic cell lysates by an active, recombinant kinase. To identify consensus motifs, the phosphorylation reaction is carried out in lysates that have previously been dephosphorylated, followed by trypsin digestion, phosphopeptide enrichment, and MS/MS analysis. Like M3, this approach benefits from the use of full-length protein substrates. However, because it is often difficult to fully dephosphorylate the lysates, the background signal may be high using this approach. Recently, Chou and colleagues described an interesting solution to this problem (Chou et al., 2012). Their approach, termed Proteomic Peptide Library (ProPeL), relies on exogenous expression of eukaryotic kinases in *E. coli*. Because *E. coli* express only two Ser/Thr kinases, ProPeL is expected to exhibit a very low background signal when examining eukaryotic Ser/Thr kinases. As a consequence, Ser/Thr phosphorylation events mediated by the kinase-of-interest should be more readily detectable. Using this approach, which uses the bacterial proteome as surrogate substrates for the kinase-of-interest, the authors accurately identified motifs for two Ser/Thr kinases, PKA and Casein Kinase II, suggesting that the use of bacterial proteins does not alter the substrate preferences of these kinases. Though it is well known that the activity of many eukaryotic kinases is poor in bacteria, the use of bacterial lysates with recombinant eukaryotic kinases could facilitate motif discovery using MS/MS-based approaches in the future.

Another attractive solution to this problem is the use of the as-kinases pioneered by Kevan Shokat's group (Bishop et al., 2001; Koch and Hauf, 2010). Analog sensitive kinases are generated by incorporating a functionally silent active site mutation into the kinase-of-interest, allowing the mutant enzyme to utilize an ATP analog that contains a bulky substituent at the N6 position (e.g., N6-benzyl-, N6-cyclopentyl-, or N6-phenylethyl-ATP). This is usually achieved by converting a bulky Met, Leu, Phe, or Thr residue in the ATP binding pocket to a smaller residue, such as Gly or Ala. Because the bulky substituent on the ATP analog cannot be used by either the wild-type version of the kinase or by the majority of other cellular kinases, incubation with the ATP analog containing radioactive phosphate at the γ-position is expected to label only direct substrates of the as-kinase. Subsequent analysis by 2D-PAGE and MS permits the identification of direct substrates. Variations on this approach, for example through the incorporation of a thiol group into the γ phosphate of the ATP analog (e.g., via [γS]-ATP^4^), also allow direct as-kinase substrates to be identified through thiol-dependent enrichment and MS/MS-based analysis. Based on the substrates and phosphosites identified through these analyses, a consensus phosphorylation motif for the as-kinase can be derived. It should be noted that, because the ATP analogs are not cell permeable, this approach is generally restricted to cell lysates. However, as-kinase-specific inhibitors, such as a series of 4-amino-1-tert-butyl-3-phenylpyrazolo[3,4-d]pyrimidine derivatives, can be used to specifically inhibit as-kinases in cells. In this way, indirect identification of as-kinase substrates can be achieved (though, as demonstrated by Bodenmiller et al., this approach may also identify substrates whose phosphorylation status is not directly modulated by the as-kinase Bodenmiller et al., 2010). Recently, Zhang and colleagues used a structure-guided approach to develop a series of potent inhibitors that target of a variety of as-kinases (Zhang et al., 2013). Though, to date, as-kinase variants and corresponding ATP analogs have been generated for only a subset of the kinases in the human kinome, the majority of kinases in the human kinome contain a bulky “gatekeeper” residue in the ATP binding pocket. Therefore, in theory, this approach is generally applicable to a large number of kinases.

Once a consensus motif is identified, it is possible to make predictions about which kinases are responsible for the phosphorylation event. Indeed, by combining information about consensus phosphorylation motifs with phosphosites identified by MS/MS analysis, several groups have developed computational methods to predict which kinase(s) is likely to phosphorylate a given site *in vivo* (Linding et al., 2007, 2008; Song et al., 2012; Newman et al., 2013). Though many of the early KSR prediction methods utilized sophisticated search algorithms, such as position-specific scoring matrices, neural networks, or support vector machines, to scan the primary amino acid sequence of a given protein for the motif-of-interest (Yaffe et al., 2001; Brinkworth et al., 2003; Blom et al., 2004; Hjerrild et al., 2004; Kim et al., 2004; Xue et al., 2005, 2006; Wong et al., 2007), these algorithms were generally more successful at identifying putative *in vitro* KSRs than they were at accurately predicting KSRs that actually occur inside the cell. This is due, in large part, to lack of contextual information about the kinase-substrate pair (Linding et al., 2008; Song et al., 2012). Therefore, in addition to consensus phosphorylation motifs, more recent platforms also integrate GO data, such as information about protein expression, subcellular localization, and protein–protein interactions, to improve the predictive power of the algorithms (Linding et al., 2007, 2008; Song et al., 2012; Newman et al., 2013). In this way, extensive phosphorylation networks have been constructed that connect cellular substrates and/or phosphosites to their immediate upstream kinase(s). Though still in their infancy, the utility of these methods has grown considerably in recent years. For instance, using current data about validated *in vivo* KSRs, the true positive rate of the NetworKIN algorithm, which pioneered the use of consensus motifs (determined by scanning peptide arrays) with contextual information about kinase-substrate pairs in cells, is 0.76% (48 true positives out of a total 6338 phosphosite identifications) (Linding et al., 2007; Newman et al., 2013). Meanwhile, more recent approaches, such as the *in vivo* group-based prediction system (iGPS) (Song et al., 2012) and the CEASAR approach (which Connects Enzymes And Substrates at Amino acid Resolution) perform markedly better, with the latter exhibiting a true positive rate of 17.2% (758/4417) (Newman et al., 2013). Though the predictive power of these approaches is currently limited by several parameters, including the availability of GO data, *in vivo* phosphosites, and information about consensus phosphorylation motifs, the high resolution maps that they generate have the potential to provide a wealth of information about the organization of cellular phosphorylation networks. This information will be invaluable for identifying those KSRs that are likely to occur inside the cell. Importantly, they also provide a framework for identifying points of signal integration between distinct pathways.

## Regulation of phosphorylation-dependent signaling pathways

In addition to information about the sites on a substrate that *can* be phosphorylated by a given kinase (or dephosphorylated by given phosphatase), it is important to know *when* and *where* phosphorylation is likely to occur inside the cell. Such information can provide key insights into the physiological consequences of phosphorylation. Indeed, the tight spatial and temporal regulation of cellular phosphorylation networks is believed to underlie the specificity of cellular signal transduction. Therefore, in order to gain a more comprehensive understanding of dynamic phosphorylation networks, the static maps of phosphorylation networks described in the previous section will need to be supplemented with information about the spatiotemporal regulation of the protein kinases and phosphatases within these networks.

Traditionally, this has been achieved by examining changes in the phosphorylation state of representative cellular substrates before and after stimulation. Changes in the phosphorylation status of these proteins are typically determined using two complementary approaches. In the first approach, cellular proteins are isolated by either subcellular fractionation or immunoprecipitation so that their phosphorylation status can be probed using one of the phosphodetection methods discussed above (e.g., 2D-PAGE, MS/MS or western blotting using phosphospecific antibodies). While careful experimental design and the use of general phosphatase and/or kinase inhibitors allows for decent temporal resolution using this approach, its spatial resolution is often poor because cellular architecture is disrupted during the lysis procedure. Moreover, since a large number of cells are required to obtain enough protein for analysis, this approach lacks single cell resolution, potentially masking subtle, yet important, differences between individual cells. The other approach, immunofluorescence using phosphospecific antibodies, offers excellent spatial resolution and can provide insights into cell-to-cell differences within a given population. However, because the cells must be fixed prior to analysis, this approach suffers from poor temporal resolution. Importantly, neither approach is able to examine changes in phosphorylation in living cells. Therefore, these methods offer only a “snapshot” of the dynamic changes in kinase and phosphatase activities that characterize most phosphorylation-dependent signal networks under physiological conditions.

### Genetically-targetable biosensors to track kinase activity profiles in living cells

**Figure 4:**
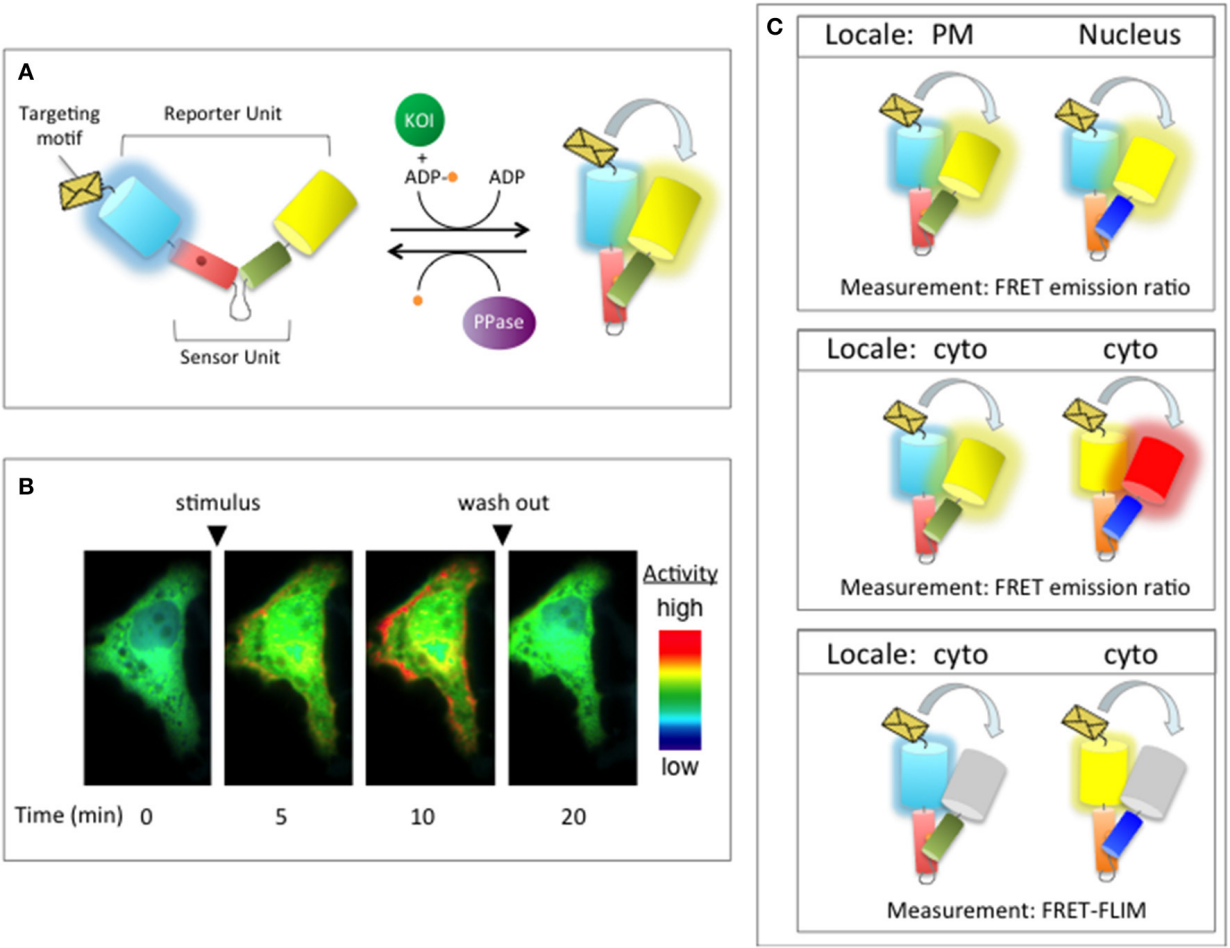
**Genetically-targetable FRET-based biosensors. (A)** Design of a FRET-based kinase activity reporter based on an engineered molecular switch. In this design, the sensor unit is composed of a PAABD (red cylinder) tethered to a substrate domain (green cylinder) that is specifically phosphorylated by the kinase-of-interest (KOI; green circle). The sensor unit is sandwiched between the reporter unit, which is comprised of two FPs that are able to undergo FRET [e.g., CFP (cyan cylinder) and YFP (yellow cylinder)]. A targeting motif (orange envelope) is used to direct the reporter to distinct subcellular regions. In the unphosphorylated state, the FPs are far removed from one another and, therefore, do not undergo FRET. An increase in the activity of the KOI leads to a phosphorylation-dependent conformational change that alters the distance and/or orientation of the FPs, increasing FRET between them. Phosphatase (PPase; purple oval)-mediated dephosphorylation of the reporter switches it back to the open conformation, reducing FRET. **(B)** Pseudocolor images of a live cell imaging experiment. In this experiment, a time-dependent increase in the FRET emission ratio (YFP FRET/CFP) is observed following stimulation with a pharmacological activator (stimulus). Upon removal of the stimulus (wash out), the emission ratio returns to basal levels. Warmer colors represent high activity while cooler colors indicate low activity. **(C)** Several ways in which genetically-targetable biosensors can be used to monitor real-time changes in the activity profiles of two or more signaling enzymes in the same cell. Top panel: Activity reporters that utilize the same FP FRET pair can be monitored simultaneously in the same cell provided that each biosensor is targeted to a distinct subcellular locale, such as the plasma membrane (PM) and the nucleus. Middle and bottom panels: To track the activities of two or more signaling enzymes in the same subcellular region, activity reporters that utilize spectrally distinct FP FRET pairs (e.g., CFP/YFP and YFP/RFP) can be used (middle panel) or alternative fluorescence imaging techniques, such as FRET-fluorescence lifetime imaging (FRET-FLIM), which only measures changes in emission of the donor fluorophore, can be employed (bottom panel). Cyto, cytoplasm; PM, plasma membrane.

In order to study the activity profiles of cellular kinases and phosphatases in single, living cells with high spatiotemporal resolution, several groups have developed genetically-targetable FRET-based biosensors (Zhang and Allen, 2007; Herbst et al., 2009; Newman et al., 2011; Sipieter et al., 2013) (Figures 4A,B). These biosensors, which can be directed to specific subcellular regions through the incorporation of a targeting motif (e.g., a NLS) or a component of a signaling complex (e.g., a scaffold protein) (Zhang et al., 2001; Kunkel and Newton, 2014), are able to monitor real-time changes in the activity profiles of specific pools of a given kinase or phosphatase in living cells (Kunkel and Newton, 2009; Gao and Zhang, 2010). Importantly, as outlined in Section Computational Models of Phosphorylation Networks, the high spatiotemporal resolution afforded by these sensors provides kinetic data that can be integrated into computational models that provide insights into the behaviors of entire signaling networks (Saucerman et al., 2006; Violin et al., 2008; Ni et al., 2011; Greenwald et al., 2014). Moreover, with the development of bright, spectrally distinct FPs that span the visible spectrum (Day and Davidson, 2009; Sample et al., 2009; Newman et al., 2011; Ai et al., 2014) and sophisticated imaging/deconvolution protocols (Grant et al., 2008; Woehler, 2013), it is now possible to measure changes in the activity of multiple cellular signaling enzymes (e.g., two kinases or a kinase and a phosphatase) simultaneously in the same cell (Carlson and Campbell, 2009; Woehler, 2013) (Figure 4C). Such information will be particularly valuable for understanding crosstalk between distinct signaling pathways within phosphorylation networks.

FRET-based biosensors contain two basic components: (1) a “sensor unit,” which undergoes a conformational change in response to a given cellular stimulus (e.g., phosphorylation or enzyme activation) and (2) a “reporter unit,” which converts the induced conformational change into a change in FRET (Frommer et al., 2009; Newman et al., 2011) (Figure 4A). While the reporter units of most FRET-based kinase and phosphatase reporters utilize a FP FRET pair consisting of cyan FP (CFP) and yellow FP (YFP) color variants, other FP combinations, such as green/red FP (GFP/RFP), yellow/orange FP (YFP/OFP), YFP/RFP and CFP/RFP, have also been used successfully (Sample et al., 2009; Newman et al., 2011; Day and Davidson, 2012; Lam et al., 2012; Ai et al., 2014). The continued refinement of the photophysical properties of FPs for FRET-based applications will be critical for the development of future biosensors. As alluded to above, this will also facilitate the development of orthogonal activity reporters that can be used to monitor more than one signaling enzyme in the same cell (Figure 4C). In this respect, the development of FPs that exhibit a large Stokes shift, such as mAmetrine (Ai et al., 2008) and T-Sapphire (Zapata-Hommer and Griesbeck, 2003), hold great promise for multicomponent FRET imaging (Carlson and Campbell, 2009).

While the “reporter unit” relies upon the FRET efficiency between the FP FRET pair, the molecular switch utilized by the “sensor unit” can take several different forms. The primary consideration when developing a molecular switch is that it promotes a conformational change in response to the cellular parameter under study. For instance, some FRET-based biosensors are designed to monitor the conformational state of the signaling enzyme itself. Indeed, kinase activation sensors, which directly monitor the activation of a kinase-of-interest, have been used to study the spatiotemporal regulation of several important kinases, including extracellular-regulated kinase 2 (ERK2) (Fujioka et al., 2006), MAP kinase-activated protein kinase 2 (MK2) (Neininger et al., 2001), Akt (Yoshizaki et al., 2006; Ananthanarayanan et al., 2007; Calleja et al., 2007), focal adhesion kinase (FAK) (Ritt et al., 2013), CaMKII (Erickson et al., 2011), and phosphoinositide-dependent kinase 1 (PDK1) (Calleja et al., 2007; Gao et al., 2011). For instance, this approach was recently used to monitor the activation of PDK1 in different membrane microdomains. To this end, Gao et al. sandwiched full-length PDK1 between enhanced CFP (ECFP) and the YFP variant, mCitrine (Gao et al., 2011). The resulting PDK1 activation reporter (PARE) was localized to either raft or non-raft regions of the plasma membrane using targeting motifs derived from Lyn and K-Ras, respectively, and used to track changes in PDK1 activation following stimulation with the growth factor, platelet-derived growth factor (PDGF). Interestingly, these studies demonstrated that, following PDGF stimulation, PDK1 is activated in raft, but not in non-raft, regions. The differential regulation of PDK1 appears to be dependent on the presence of the lipid phosphatase PTEN, which dephosphorylates phosphatidylinositol (3,4,5) triphosphate (PIP_3_) required for PDK1 activation. Because PDK1 is a master regulator of many AGC family members, including Akt, PKA, SGK and several PKC isoforms, these results have important implications for understanding the regulation of PDK1-dependent signaling networks (Arencibia et al., 2013).

In addition to biosensors designed to monitor kinase *activation*, many biosensors have also been developed to monitor changes in kinase *activity*. Unlike the activation reporters discussed above, which exhibit a linear relationship between the signal response and the activation “event,” kinase activity reporters benefit from enzymatic amplification of the signal. This is because multiple reporter species can be phosphorylated by a single activated kinase. This feature stems from the design of the sensor unit. Indeed, the sensor units of all currently available kinase activity reporters utilize an engineered molecular switch based upon a modular design (Zhang and Allen, 2007; Mehta and Zhang, 2011; Newman et al., 2011) (Figure 4A). Accordingly, a consensus phosphorylation site specific for the kinase-of-interest serves as the “substrate domain” while a PAABD specific for the phosphorylated form of the substrate functions as the “switching segment.” The substrate domain and the switching segment are typically joined together by a flexible linker and sandwiched between a FP FRET pair, such as CFP and YFP. Whereas the length of the linker and the choice of FRET pairs influence the dynamic range of the reporter (Komatsu et al., 2011; Belal et al., 2014), the choice of the substrate domain and PAABD contribute to its specificity and reversibility, respectively. The reversibility of the reporter is critical for monitoring kinase attenuation, which provides a clearer picture of kinase regulation within intact signaling networks. For instance, although the first generation A-kinase activity reporter, AKAR1, enabled kinetic analysis of subcellular PKA activity induced by the β-AR agonist, isoproterenol, this sensor was unable to report the attenuation of PKA activity following receptor desensitization because its response was essentially irreversible inside cells (Zhang et al., 2001). This is likely due to the fact that the 14-3-3τ PAABD utilized by AKAR1 binds the phosphorylated form of the substrate domain very tightly, preventing cellular phosphatases from gaining access to the substrate region as PKA activity declines. Therefore, in order to visualize both increases and decreases in PKA activity, a second generation PKA reporter, AKAR2, was constructed by replacing 14-3-3τ with the weaker-binding PAABD, forkhead-associated 1 (FHA1) (Zhang et al., 2005a). While the activation profile of AKAR2 is similar to that observed for AKAR1, the former is also readily reversible following removal of agonist or treatment with the PKA inhibitor, H89.

The basic modular design described above has been used to create activity reporters for a number of protein kinases, including PKA, protein kinase C (PKC), ataxia telangiectasia mutated (ATM), Akt, Abl, Src, aurora kinase B, ERK, c-Jun N-terminal kinase (JNK), cyclin-dependent kinase 1 (CDK1), AMP-activated protein kinase (AMPK), and the epidermal growth factor receptor (EGFR) (Zhang and Allen, 2007; Newman et al., 2011; Tsou et al., 2011; Belal et al., 2014). Using these reporters, researchers have uncovered important details about both the kinetics and the spatial distribution of endogenous kinase action in a variety of cellular contexts (Zhang et al., 2005a; Zhang and Allen, 2007; Erickson et al., 2011; Gao et al., 2011; Komatsu et al., 2011; Mehta and Zhang, 2011; Newman et al., 2011; Tsou et al., 2011; Arencibia et al., 2013; Ritt et al., 2013; Belal et al., 2014). However, despite their unique ability to track kinase activity in real time and at single cell resolution, to date, kinase activity reporters are available for <3% of the 518 human kinases in the human kinome. This is due, in large part, to limited information about the substrate specificity of most human kinases. Indeed, this is one of the primary obstacles to the large-scale development of kinase activity reporters. The recent identification of consensus phosphorylation motifs for 289 unique kinases (representing ~55% of the human kinome) (Newman et al., 2013), as well as the continued characterization of PAABD family members (Jin and Pawson, 2012; Reinhardt and Yaffe, 2013), will facilitate the development of a large number of novel kinase activity sensors that promise to offer important insights into the spatial and temporal regulation of cellular phosphorylation networks.

### Genetically-targetable biosensors to track phosphatase activity profiles in living cells

In addition to information about kinase regulation, a comprehensive map of phosphorylation networks will also require an understanding of the dynamic regulation of protein phosphatases. However, until recently, a general design for phosphatase activity reporters had not been described. To address this issue, we engineered a phosphatase activity sensor designed to measure the activity of the Ca^2+^/calmodulin-regulated Ser/Thr protein phosphatase, CaN (Newman and Zhang, 2008; Mehta and Zhang, 2014). This reporter, termed CaN activity reporter 1 (CaNAR1), utilizes an intrinsic molecular switch based upon dephosphorylation-induced conformational changes within the regulatory region of NFAT1 (Okamura et al., 2000). Upon dephosphorylation, CaNAR1 exhibits an increase in its emission ratio that is dependent on CaN activity. Importantly, because the regulatory region of NFAT1 is hyperphosphorylated in resting cells by cellular kinases such as p38 and the constitutively-active kinase, casein kinase 1 α (CK1α), CaNAR1 does not require activation of additional kinases to put it into a “dephosphorylation competent” state. This feature ensures that the cellular environment remains relatively unperturbed prior to Ca^2+^ stimulation. This and other design features utilized by CaNAR1 should be generally applicable to other protein phosphatases as specific molecular switches are identified or engineered. Thus, as a prototype phosphatase activity sensor, CaNAR1 lays a foundation for studying the targeting and compartmentalization of protein phosphatases within the cellular environment.

### Computational models of phosphorylation networks

The development of novel biosensors will enable the activity profiles of a large number of protein kinases and phosphatases to be measured within the cellular environment, providing the experimental information necessary to build detailed computational models of phosphorylation networks. Such approaches will be important in order to gain a systems-level understanding of the complex regulation of phosphorylation networks in response to various cellular stimuli. To this end, quantitative FRET-based imaging data has been incorporated into mathematical models of phosphorylation networks based on ordinary differential equations (Saucerman et al., 2006; Violin et al., 2008; Ni et al., 2011; Song et al., 2012). One of the primary goals of this approach is to determine the relative contributions of multiple feedback and feed-forward loops in producing the tight spatiotemporal control exhibited by many signal transduction pathways. Not only does this tact help to uncover details about the behavior of individual components within a given signaling network, but it can also provide crucial insights into how information is propagated throughout the entire system. Because these types of questions are difficult (if not impossible) to answer using experimental approaches alone, computational methods can provide a more comprehensive view of signaling networks that will ultimately promote a better understanding of cellular signaling at the systems level. For instance, computational models have been developed to study the impact of signaling enzymes in diverse cellular processes, including PKA-mediated phosphorylation gradients in cardiomyocytes (Saucerman et al., 2006), hippocampal neurons (Neves et al., 2008), and pancreatic β-cells (Ni et al., 2011). Similar models have also been constructed to examine the mechanisms by which the activities of Ca^2+^/calmodulin-dependent signaling enzymes, such as CaMKII and CaN, are differentially regulated in response to dynamic changes in intracellular Ca^2+^ concentrations (Song et al., 2008).

In addition to computational models based on fluorescence imaging data, the advent of quantitative MS/MS methods, such as SILAC and iTRAQ, has also permitted the development of computational models that focus on global changes in phosphorylation profiles (Kozuka-Hata et al., 2012). While models based on data obtained using fluorescent biosensors can best be described as kinetic and/or stochastic because they are built upon detailed kinetic information about the behavior of each component in the system (e.g., individual kinase and phosphatase activities), models based on quantitative MS/MS data are best characterized as discrete models because they describe changes in the general profile of an entire population or system (Wu et al., 2009). For instance, computational methods based on self-organizing maps (Zhang et al., 2005b), partial least squares regression analysis (Wolf-Yadlin et al., 2006; Kumar et al., 2007), Bayesian network modeling (Bose et al., 2006; Guha et al., 2008), and numerical modeling (Tasaki et al., 2006) have been developed. More recently, Tian and Song described a general computational framework that can be used to develop mathematical models derived from multiple quantitative phopshoproteomic data sets (Tian and Song, 2012). This model, which was used to model MAPK signaling pathways, will be particularly useful for modeling highly time-resolved phosphoproteomic data sets, such as the one reported by Oyama et al. (2009). In this study, the authors used SILAC and pTyr-enrichment to construct a detailed kinetic analysis of the Tyr phosphoproteome following EGFR activation in human epithelial A431 cells. These studies, which measured relative levels of Tyr phosphorylation at 0.5, 2, 5, 10, 15, 20, 25, and 30 min following EGFR activation, identified both transient and sustained changes in pTyr levels among a collection of 77 different cellular proteins.

Importantly, with continued advances in biosensor development and quantitative MS/MS methodologies, the quality and depth of information that can be incorporated into models of phosphorylation networks will continue to increase, promoting a more quantitative understanding of the molecular mechanisms that influence signaling dynamics inside the cell. Such quantitative information will be critical if we are to truly understand the functional interactions that occur between individual signaling enzymes to drive complex cellular behaviors.

## Conclusion and perspective

Phosphorylation-dependent signaling networks underlie diverse cellular processes, including metabolism, cell cycle progression, the immune response, and cell migration. Recently, the emergence of several novel technologies has provided a truly systems-level view of dynamic phosphorylation networks. Together, these technologies are beginning to provide us with a clearer picture about (1) which cellular proteins are phosphorylated in response to a given stimulus and/or in a particular cellular context, (2) where on the protein the phosphorylation event occurs, (3) which kinases and phosphatases are mediating phosphorylation/dephosphorylation of the phosphosite, and (4) how these modifying enzymes are regulated inside the cell. In the future, it will be important to expand this knowledge-base through (1) systems-wide profiling of phosphoproteomes under different cellular conditions and disease states using quantitative MS/MS and 2D-DIGE; (2) the identification of additional KSRs [and their relatively under-studied counterparts, phosphatase-substrate relationships (PSRs)] using “whole-proteome” microarrays; (3) the development of novel fluorescent biosensors and orthogonal imaging modalities; and (4) the integration of these data into predictive models of phosphorylation networks. Though much work remains to be done, these goals appear to be within reach. The next challenge will be to gain a detailed understanding of the functional consequences of specific phosphorylation events and to integrate this information with other resources, such as protein–protein interaction data sets, global expression profiles, and metabolomics data (Derouiche et al., 2012; Harrold et al., 2013; Medina-Cleghorn and Nomura, 2013; Bordbar et al., 2014). Likewise, in order to understand crosstalk between different cellular signals (e.g., O-glycosylation and phosphorylation), it will be necessary to adapt the approaches developed to study phosphoproteomics to other post-translational modifications (Choudhary and Mann, 2010; Zhu et al., 2012; D'hondt et al., 2013; Sutandy et al., 2013). Together, this information will provide a comprehensive view of the organization and regulation of cellular phosphorylation networks and beyond.

### Conflict of interest statement

The authors declare that the research was conducted in the absence of any commercial or financial relationships that could be construed as a potential conflict of interest.

## Footnotes

^1^For simplicity, unless otherwise noted, we will focus primarily on kinase-mediated phosphorylation of cellular substrates for the remainder of this discussion; however, it is important to note that the same principles apply for phosphatase-mediated dephosphorylation of cellular proteins.

^2^The “top-down” and “bottom-up” approaches described here should not be confused with similar terms used to describe MS-based proteomic analysis of intact proteins (“top-down”) or peptide fragments (“bottom-up”).

^3^It should be noted that MS/MS can also be achieved “in time” using a single mass analyzer with multiple separation steps being instituted at different times.

^4^[γS]-ATP can be utilized by most protein kinases, but not by most other cellular ATPases (Koch and Hauf, 2010).
